# Quality assessments for cancer centers in the European Union

**DOI:** 10.1186/s12913-016-1738-2

**Published:** 2016-09-07

**Authors:** Anke Wind, Abinaya Rajan, Wim H. van Harten

**Affiliations:** 1 Division of Psychosocial Research and Epidemiology, The Netherlands Cancer Institute-Antoni van Leeuwenhoek Hospital, Plesmanlaan 121, 1066 CX Amsterdam, The Netherlands; 2 Department of Health Technology and Services Research, University of Twente, P.O. Box 217, 7500 AE Enschede, The Netherlands; 3 CEO Rijnstate Hospital, Wagnerlaan 55, 6815 AD Arnhem, The Netherlands

**Keywords:** Quality assessments, Cancer centers, Patient safety, European Union

## Abstract

**Background:**

Cancer centers are pressured to deliver high-quality services that can be measured and improved, which has led to an increase of assessments in many countries. A critical area of quality improvement is to improve patient outcome. An overview of existing assessments can help stakeholders (e.g., healthcare professionals, managers and policy makers) improve the quality of cancer research and care and lead to patient benefits. This paper presents key aspects of assessments undertaken by European cancer centers, such as: are assessments mandatory or voluntary? Do they focus on evaluating research, care or both? And are they international or national?

**Methods:**

A survey was sent to 33 cancer centers in 28 European Union member states. Participants were asked to score the specifics for each assessment that they listed.

**Results:**

Based on the responses from 19 cancer centers from 18 member states, we found 109 assessments. The numbers have steadily increased from 1990’s till 2015. Although, a majority of assessments are on patient-care aspects (*n* = 45), it is unclear how many of those include assessing patient benefits. Only few assessments cover basic research. There is an increasing trend towards mixed assessments (i.e., combining research and patient-care aspects)

**Conclusions:**

The need for assessments in cancer centers is increasing. To improve efforts in the quality of research and patient care and to prevent new assessments that “reinvent the wheel”, it is advised to start comparative research into the assessments that are likely to bring patient benefits and improve patient outcome. Do assessments provide consistent and reliable information that create added value for all key stakeholders?

**Electronic supplementary material:**

The online version of this article (doi:10.1186/s12913-016-1738-2) contains supplementary material, which is available to authorized users.

## Background

Cancer Centers (CCs) in Europe, are located in complex organizational and regulatory environments and are increasingly under pressure to deliver high-quality services and be transparent about it [1]. As a consequence of this, there is an increasing emphasis on quality and safety improvement initiatives [2]. Patients and payers increasingly demand proof of guaranteed safety and quality of services. Cancer care activities lead to a steadily growing financial claim on national and regional health systems leading to concerns on sustainability and value for money, especially at a time of austerity measures and deficits in public budgets [3, 4]. This has led to additional need for transparency on quality matters and performance issues [5].

Determining what is quality and safety of care is complex as it can reflect the combined perspectives of policy makers, purchasers, payers, healthcare professionals, researchers and patients [6]. The complexity of healthcare systems and the unpredictable nature of health care adds to this difficulty [7]. Setting and applying clear performance standards through regulatory mechanisms, such as licensing, certification, and accreditation, is crucial to ensure patient safety [8].

CCs go through several assessments on their performance and quality, assessments being defined as: “A system for evaluating performance, as in the delivery of services or the quality of products provided to consumers, customers, or patients” [9]. Its nomenclature extends to accreditation, certification, performance review, (performance) evaluation and others. This study uses the term assessments as it includes all of the above mentioned terms. So far an overview of the assessments on a European level does not exist. A recent study among Canadian Oncologists by Lim et al. [10] shows that one of the reasons for them not participating in this type of quality improvement initiatives is the lack of knowledge about on-going initiatives. This example shows the relevance of obtaining an overview of assessments.

This article presents key findings from a survey that was conducted with CCs in the European Union. The goal was to obtain an overview of existing assessments in terms of whether they are: mandatory or voluntary; focused on evaluating research or patient care or both; regional, national and/or international. An example from the Netherlands [11] shows that hospitals spend between 40.1 to 82.3 million Euros on quality assessments in 2014. This study showed that much of the information gathered through these assessments is, however, recorded twice, inefficiently and is accompanied by bureaucracy [11]. Unfortunately there is limited evidence on the added value of these (organizational) assessments for patient care or patient outcomes, primarily due to methodological issues related to limited insight into the mechanisms through which these exert their effects. Though very relevant, that is not the object of this overview.

The rationale for this study was originally to provide input for the BENCH-CAN project [12]. The BENCH-CAN project [12] aims at benchmarking comprehensive cancer and yield best practice examples at eight European CCs in order to contribute to improvement of multidisciplinary patient treatment. One of the objectives of the BENCH-CAN project is: To collect, compare and align, by consensus formation, the standards, recommendations and accreditation criteria of comprehensive cancer care adopted in selected European countries representatives of different geographic areas (North-Western Europe; Southern Europe; Central-Eastern Europe). Because of the potential to inform decision makers about existing assessments so that they can take some steps towards regulating these as well as minimizing the related bureaucracy, it was decided to expand the study to other CCs than just the BENCH-CAN pilot sites.^.^ Organizations conducting these assessments and (also non EU) CCs can gain better understanding of what type of assessments are currently undertaken in view of growing interest in cooperation in international research consortia [13, 14].

### The context of European cancer centers

**Fig. 1:**
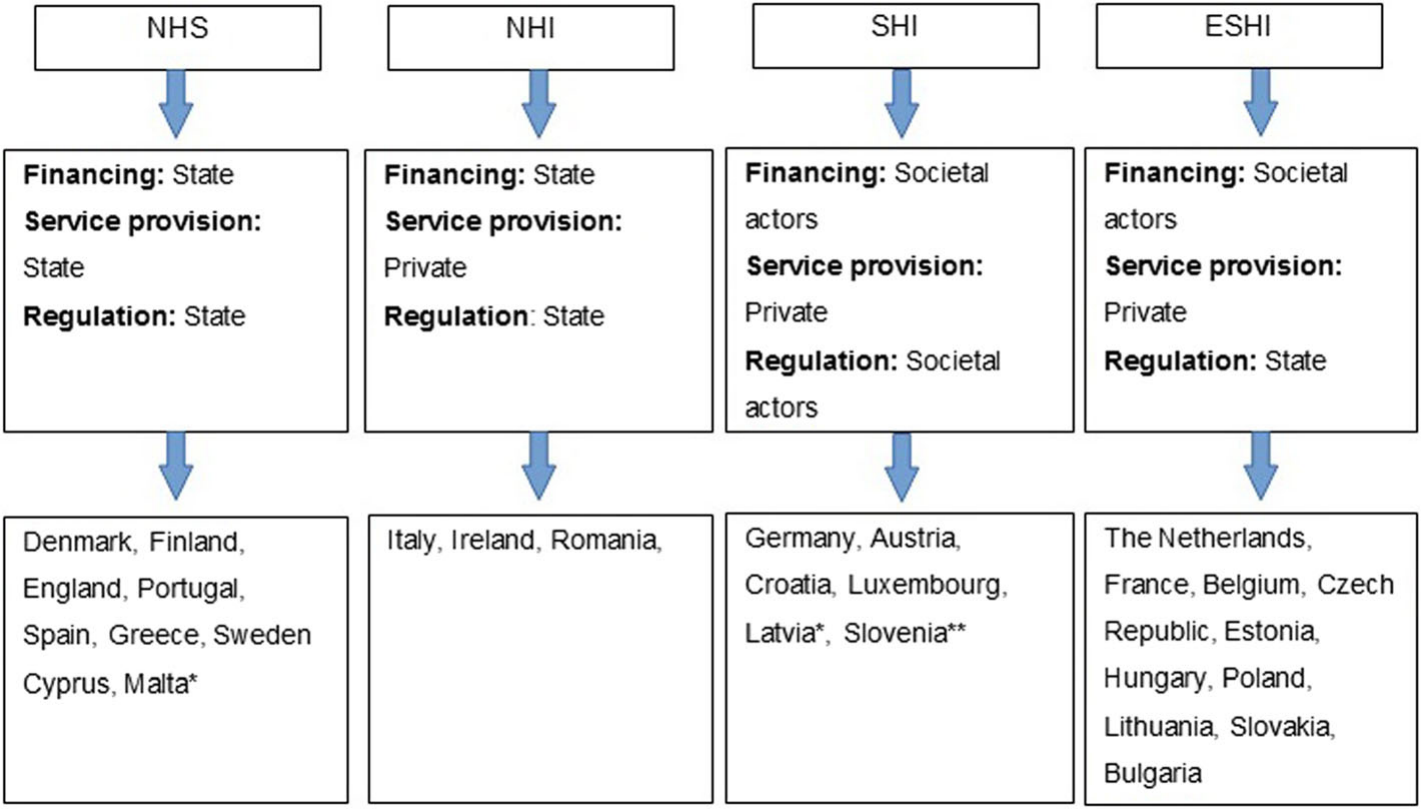
Overview of typology of health systems in the EU. * Malta and Latvia have mixed public/private service provision. ** Slovenia conflicts with the logic of the RW typology as societal actors are in charge of regulation and financing, but service provision lies predominantly in the hands of state actors. Slovenia is, however, gradually evolving into a SHI

Assessments are contextual, and so, first there is a need to understand the type of health system in which the CCs operate. Health systems in the EU can be described in different ways. For this article, the typology developed by Rothgang et al. [15] and Wendt et al. [16] was used, which suggests four types of health systems: the National Health Service (NHS), National Health Insurance (NHI), Social Health Insurance (SHI) and the Etatist Social Health Insurance (ESHI). Three dimensions distinguish each of these systems: financing, service provision, and regulation [17]. According to this classification scheme each dimension can be dominated by state (government), societal (for example NGO’s, consultancy agencies or research institutes), or private actors (see Fig. 1). The US system has a mix of characteristics of those systems; however, unique about the US system in the world is the dominance of the private for profit actors in all three dimensions over the public sector (state/government and societal/ non-governmental) [18].

## Methods

### Survey

A survey (see Additional file 1) was sent initially to the BENCH-CAN pilot sites. After the decision to expand the study, the survey was sent to one cancer center in each of the EU member states with the exception of Belgium, Austria and the UK where 2 cancer centers were contacted. This was due to the lack of response within the given time-frame from the first contacted center. A second center was contacted in each of these countries. In total the survey was sent to 33 cancer centers in the 28 EU countries. Ethics committee approval was deemed irrelevant for this study. For some member states, CCs could not be easily identified and so, other organizations dealing with cancer care and/or research were contacted. CCs were identified through the European Society for Medical Oncology (ESMO) [19] and the Organisation of European Cancer Institutes (OECI) [20]. The survey was addressed to the lead administrative person in each institute. Participants were asked to describe several topics for each assessment that they listed: (i) the name of the assessment body (i.e., organization that performed the assessment) (ii) whether the body was public or private; (iii) if the assessment was mandatory or voluntary; (iv) the level (i.e., regional/national/international) at which the assessment was performed; (v) if the assessment focused on research, patient care aspects or a mix of standards (vi) the frequency of the assessment; (vii) if the assessment led to keeping/losing operating license and/or public funding and (viii) the year in which the assessment was first performed.

### Data management and inclusion/exclusion criteria

Among the 28 EU member states in which CCs were asked to participate, data were received from 18 member states i.e., one cancer institution per member state (64 %), with the exception of Italy (two cancer institutions). Not all surveys were filled out correctly and some were missing data. A follow up was done by e-mail or phone with all respondents to clarify the answers. Two researchers inspected the data and excluded the listed assessments that did not fit the inclusion criteria. The inclusion criteria for the assessments were: the assessment had to assess cancer care, cancer research or a combination of both. All assessments that did not fit these criteria were excluded from the study. Eligible assessments were divided into three categories: clinical/patient care oriented assessments; research oriented assessments; and assessments that are oriented at a combination of care and research. Clinical/Patient care oriented assessments focus on the care delivered by Cancer Centers, Research oriented assessments focus solely on research performed at Cancer Centers, while combination oriented assessments focus on a comprehensive assessment of both the care delivered as the research performed in the CC (only applicable in centers were both activities are fully developed). A content analysis was performed. This method enables a more objective evaluation than comparing content based on the impressions of a reader and simplifies the detection of trends. This analysis was executed by converting the different items of the survey (public or private; mandatory or voluntary; regional/national/international; focused on research, patient care aspects or both; the frequency; if the assessment led to keeping/losing operating license and/or public funding and the year in which the assessment was first performed) into variables in excel. By dividing the data into the variables, a structured overview of assessment characteristics was obtained. This overview enabled the researchers to investigate trends in assessments and possible relationships between types of assessment and health systems. Two researchers independently examined the data to check for Inter Rater Reliability. The analysis of findings includes only programs that completed the survey. Validity of the data was verified by checking the assessment body in an online search and by asking the participating CCs to double check the data provided. The full list of included assessments was circulated amongst the respondents for final data validation.

## Results

### Nature and scope of assessments

Based on the responses, we found 109 known cancer related quality assessments in total in 19 EU member states (see Additional file 2). The majority of the assessments focus on patient-care aspects (*n* = 45), such as waiting and throughput times, patient participation and patient satisfaction followed by the mixed assessments that focus on patient care as well as research aspects (*n* = 37). In those mixed assessment especially organizational aspects of care and research such as multidisciplinary harmonization / integrated care and scientific interaction and integration receive emphasis, whereas pure research oriented assessments, which are the least in number (*n* = 27), are directed towards research outcomes such as number of publications. The majority of patient care oriented assessments are reported to be mandatory. Mixed assessments are more voluntary.

The majority of assessments (*n* = 62) is done at the national level (performed by national government sponsored federal agencies or performed by national ‘bodies’ unaffiliated with governments but with assessment authority), followed by 34 assessments that are known to be operational at an international level (performed by international assessment agencies). Some assessments are implemented at a national level, but are also operational at an international level, these have been counted as national. There are only a handful of regional assessments (*n* = 9) such as in Estonia and in Finland (see Table 1). Almost all mandatory assessments are national and are mainly related to keeping license and/or receiving public funding. In contrast, most voluntary assessments are international, and rather aim at quality improvement and are seldom directly tied to licensing or funding.

**Table 1:** Level of assessments per country

EU member state	International	National	Regional
Austria	-	6	-
Croatia	-	1	-
Czech Republic	2	6	-
Denmark	1	-	-
Estonia	-	5	2
Finland	4	3	2
France	1	2	-
Germany	-	3	-
Hungary	2	1	-
Ireland	-	1	-
Italy	6	-	1
Lithuania	1	6	-
Netherlands	3	2	-
Poland	-	4	2
Portugal	2	3	-
Slovenia	12	8	-
Spain	2	3	2
United Kingdom	2	8	-
TOTAL	38	62	9

### Trend of assessments

**Fig. 2:**
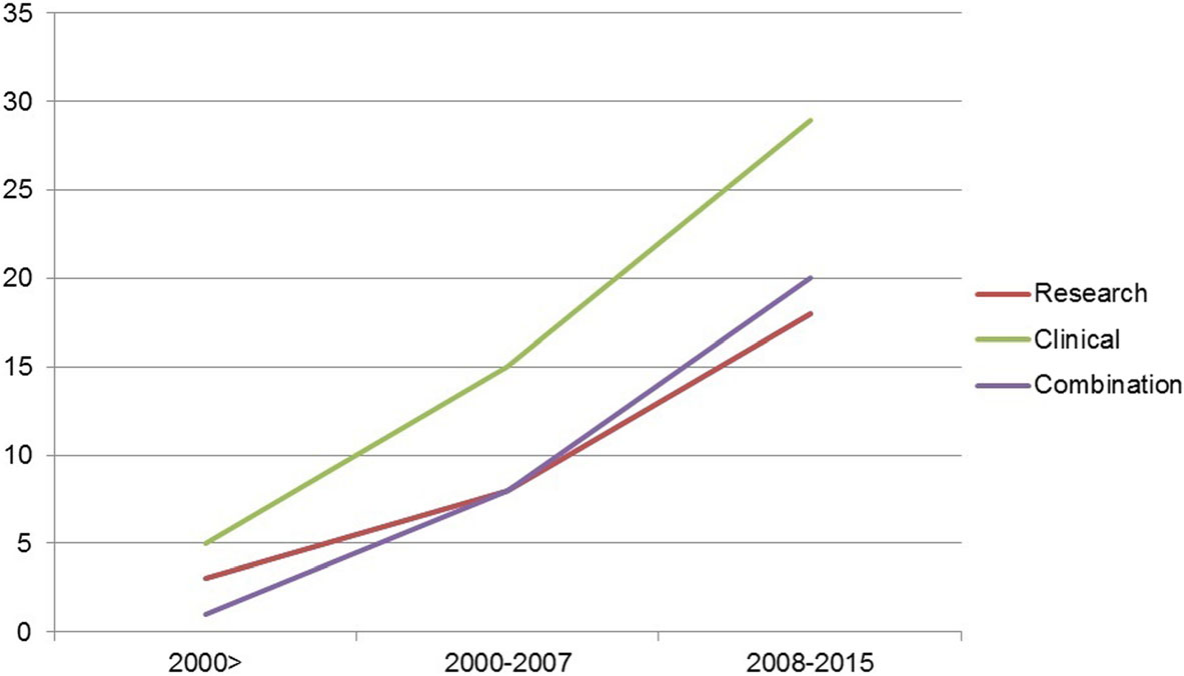
Trends in the number and types of assessments

Respondents were asked in which year the first assessment for the assessments began (see Additional file 3). For some this can be easily identified, but for a majority it is difficult to date precisely. The graph in Fig. 2 shows a cumulative presentation of the trends in the number and types of assessments. It suggests that:

- The numbers of assessments have steadily increased from the 1990’s till 2015.
- In over the past two decades, there has been most increase in patient care assessments, followed by the mixed assessments of patient care and research aspects. The rise in pure research assessments has been the least.

## Discussion

### Continuous growth of assessments in Europe and how it compares to the US

The number of assessments in the EU has tripled since 2000. This shows that quality assessments in all its forms is a growing industry. It is particularly interesting to note a steady rise from 2000 to 2007, and especially following the economic crisis (2007/2008) more assessments seem to have cropped up. Whether this steep rise is related to the need for more accountability during and post financial crunch situations is hard to say. Although the emphasis on mandatory assessments will remain for the purpose of funding and licensing health services, voluntary assessments are equally gaining in popularity. In fact, most of the new assessments are voluntary, however, this does not exclude the pressure on CCs to participate in them. This shows that most assessments seem to be in a transition, moving from a friendly tool of self-assessment and development to a governing tool that agencies use for various purposes.

Regarding CCs, in the US there are at least three main assessments: The Joint Commission accreditation [21] for healthcare organizations and programs as a whole; The Commission on Cancer (CoC) of the American College of Surgeons for the quality of cancer care delivery [22]; and The National Cancer Institute (NCI) designation [23] for assessing excellent multidisciplinary translational cancer research programs, in which almost all leading CCs in the US participate. Europe is gradually moving towards common European assessment frameworks in order to benchmark and improve cancer research and patient care activities across the EU, but this has not been as developed as it is in the United States. It is with this intention that European Commission is allocating more budgets for research and innovation (e.g., through specific funding programs such as Horizon2020) [24] with the idea of improving EU competitiveness in excellent science [25]. However, the challenges that arise from health care being under national jurisdiction and individual responsibility of each EU member state has meant that only gradual steps towards harmonization of EU assessments have been seen so far. As healthcare is a major component of national economies (as a user of public funds but also as an investment that generates jobs, taxes and procurement opportunities for Small and Medium Enterprises) within a monetary union, increasing steps towards EU influence on these issues seems inevitable [26].

### The link between health system type and nature and scope of assessments

A link between the type of health system and the nature of the assessments is visible only in some member states. For example, in the United Kingdom where a National Health Service is being used (regulation, financing and provision by the state, see Fig. 1) a lot of mandatory, national assessments can be found. The same goes for Spain. In other countries that have an NHS model, e.g., Finland and Portugal assessments seem to be more voluntary than mandatory. Within the National Health Insurance system (regulation by the state) one would again expect a lot of mandatory and national assessments, but the opposite is the case in Italy, where a lot of international voluntary assessments are performed e.g., the Joint Accreditation Committee- International Society for Cellular Therapy and European Society for Blood and Marrow Transplantation (JACIE) [27] and International Organization for Standardization (ISO) [28] and the European accreditation by the Organization of European Cancer Institutes (OECI) [20]. But these initially voluntary assessments are sometimes mandatory for either keeping license and/or are demanded by government to maintain a Comprehensive Cancer Center status, such as in Italy. Hence, voluntary assessments end up being mandatory at some level.

In the Social Health Insurance (SHI) type, societal actors dominate healthcare regulation and financing, which is reflected in the assessments listed by the centers from Germany and Croatia e.g., in Germany accreditation of cancer care is performed by the German Cancer society [29] which is a societal actor dominated by physicians. In most Central and Eastern European countries that have an Etatist Social Health Insurance system, there is a tendency for more mandatory national assessments, while in the majority of Western Europe and Nordic countries there is tendency to participate in more voluntary international assessments. Only in few member states, did we notice regional level assessments e.g., Italy, Finland and Estonia. This can be partly explained by decentralization/devolution of powers to regions in some EU member states [30]. Evidence suggests that mandated external quality assessments are less effective than voluntary assessments because the effectiveness of accreditation is dependent on its voluntary nature, non-threatening process, and interactive process with external reviewers as a means of effecting and speeding up quality improvements [31].

### Traditional view of assessments and shifting focus

Assessments focused on research performed by CCs (such as LabQuality which checks the quality of Laboratories and BASG/AGES that looks at the quality of clinical drug trials) are still limited in Europe when compared to patient care assessments. The NCI designation program [23] in the US is one of the anchors of the nation’s cancer research effort. In order to be designated, CCs must meet specific criteria for: breadth and depth of basic cancer research; clinical cancer research; prevention, control and population/behavioral sciences research in cancer; and strength of interaction among these three major research areas. A European version of the NCI designation was not found in our study.

One of the possible reasons why patient care assessments (such as certain accreditations) are performed more often than research focused assessments is that, being an accredited center in cancer care could attract patients [32]. Additionally, in some countries, accreditation is being used as an extension of statutory licensing for institutions [33]. Therefore, care assessments such as accreditation seem of more direct importance than assessing research. Another reason is that assessing impact of research on healthcare outcomes is more difficult than assessing care outcomes [34]. In research, metric-driven indicators such as impact factors are often criticized [35] and consensus on value-based indicators is still evolving e.g., how to define success in translational research (bench to bedside and back) in terms of practice-changing innovations [36]. The awareness that alignment between research and clinical areas is essential in successful translational research [37] can explain why more mixed assessments are being introduced in the EU. This is comparable to the SPORE [38]-the Specialized Programs of Research Excellence-a cornerstone of National Cancer Institute’s efforts dedicated to capitalize on research opportunities that have the potential to change the current paradigm in the prevention, detection, diagnosis, and/or treatment of cancer. Given the amount of funding that goes into research in the EU as in the US [39], evaluating research becomes necessary. More specifically, comparative research assessments are needed to make evidence based decisions on most suitable therapies in clinical practice [40].

### Transparency

A review of accreditation and quality systems by the World Health Organization [41] shows that “the move towards statutory and governmental endorsement is associated with freer access by the public to the standards, processes and findings of accreditation”. Half of the assessment bodies make the standards/indicators used for the assessment available at little or no cost. One-third also makes full reports of individual assessments publicly available. However, several organizations are unwilling to give away their standards and norm descriptions as this serves also as a source of income and intellectual property. Other difficulties are for example the fact that in many EU member states, the assessment reports as well as the program standards are in the local language. It takes time, money and effort to accurately translate the reports into English. This makes it hard to judge assessments in terms of how each assessment can bring added value to the different stakeholders [41]. The first step in deciding the value of assessments is to make their outcomes publicly available and accessible [42] although this assumption has been challenged [43]. Next, public consultation must occur with key stakeholders to decide the parameters to assess the added value of assessments [44]. Another issue related to this is also whether the data, if made publicly available, are good enough to actually promote quality improvement and helping consumers make choices [6]. Evidence on whether the assessments undergone by CCs actually provide added value for patient care or patient outcomes is limited. Although most assessments focus on patient care aspects, it is unknown whether patient outcomes are actually improved through these assessments [45]. Evidence shows that for example patient safety can be improved if a healthcare organization undergoes licensing, certification and accreditation [7] but this is unknown for patient outcomes. Although there is no decisive evidence on the direct impact on patient outcomes, there is some indication that quality assessments such as accreditation could contribute to health outcomes. This is the case if these assessments strengthen interdisciplinary team effectiveness, communication, and enhanced use of indicators leading to evidence-based decision making [6]. This evidence is however limited and study designs are weak. A study focusing on accreditation specifically shows that a lot of information on the added value of assessments is unknown and future research should focus on: determining the impact of accreditation on patient care and outcomes; determining how best to research the validity, impact and value of accreditation processes in health care; determination of value for time and money; and determining the reliability of accreditation surveys to truly assess the quality of organizations [45].

### Strengths and limitations

This study describes the type and number of assessments at 19 cancer centers in 18 out of 28 member states of the European Union. This is the first systematic European attempt to gather data on assessments for cancer centers. The results were validated with study participants by asking them not just to confirm the data for their own cancer center but also giving them an opportunity to comment on assessments that were listed by other cancer centers in Europe. This study gives sufficient base data to start thinking about how to reduce the burden of assessments for cancer centers and how to make them more transparent and effective.

Content of these assessments (e.g., assessment reports, outcomes) were not easy to access due to language barriers (each cancer center has it in its local European language and is not always translated in English) and/or lack of publicly available information. The individuals from cancer centers who provided the data were quality managers (and/or research directors/senior executive managers) who are usually responsible for organizing and implementing assessments in their center, However, many assessments are multidisciplinary in nature, involving a wide range of staff, therefore future research should focus on validating the responses beyond quality managers. Our assumption is that non-responses may have been the result of not identifying or contacting the appropriate people, rather than reluctance to provide data and/or that formalized assessments do not exist in some member states. Another limitation regarding the year in which the assessment started is the fact that, first assessments may be considered as pilot testing rather than becoming operational. It is therefore difficult in some cases to identify the year in which the actual assessment started.

## Conclusion

There seem to be 109 assessments that CCs currently undergo in 19 EU states and the numbers keep increasing. Although there are benefits of assessments, more robust research is needed to understand their value in terms of how they improve patient quality and safety. CCs go through frequent assessments, sometimes as often as more than once a year, this can be very time consuming as well as expensive for those organizations. Rapid uptake of voluntary assessments is associated with direct financial incentives (such as linkage to core funding or reimbursement) and government encouragement. However, decision makers should regulate assessments to reduce unnecessary assessments that do not bring benefits or added value, that are bureaucratic, time-consuming and/or unaffordable by CCs. This article shows that demand for assessments is increasing and changing rapidly in terms of international assessments as well as mixed assessments of cancer research and care. Assessments must be transparent to bring credibility and accountability among stakeholders. Given the importance of quality of care, patient safety and outcome improvement in cancer care, it would be desirable to evaluate the impact of assessments in these areas. We recommend future research to go deeper into understanding process and outcome related issues; how much time does each assessment take to prepare and implement, people and money consumed, who are the peer-reviewers and what are their backgrounds, how are standards developed and revised, sources of income for assessment bodies, and last but not least does the exercise meet its objectives?

## Additional files

Additional file 1: Questionnaire Quality assessments Cancer Centers. This file contains the survey that was send to the cancer centers to obtain the data. (DOC 40 kb) Additional file 2: Table 2. Scope and nature of assessments. This table contains information regarding the Scope (patient, research, combination) and Nature (mandatory or voluntary) of the assessments per country. (DOC 49 kb) Additional file 3: Table 3. Overview of assessments including details per country. This table contains all assessments included in this study, divided per country and category (patient, research, combination). (DOC 124 kb)

## Abbreviations

CC: Cancer center
COC: Commission on cancer
ESHI: Etatist Social Health Insurance
ESMO: European Society for Medical Oncology
EU: European Union
NAPBC: National Accreditation Program for Breast Cancers
NCI: National Cancer Institute
NGO: Non-Governmental Organization
NHI: National Health Insurance
NHS: National Health Service
OECI: Organisation of European Cancer Institutes
SHI: Social Health Insurance
