# Supplementary material for: Quality assessments for cancer centers in the European Union
Source: BMC Health Serv Res. 2016 Sep 7;16(1):474. doi: 10.1186/s12913-016-1738-2 (PMC5013566; doi:10.1186/s12913-016-1738-2)
Supplement: Additional file 1: — Questionnaire Quality assessments Cancer Centers. This file contains the survey that was send to the cancer centers to obtain the data. (DOC 40 kb) [file 12913_2016_1738_MOESM1_ESM.doc]

**ANNEX-I Survey**

Name of the centre:

Name of respondent:

Job role of respondent:

1. Which of the following activities take place in your centre (in house). Please highlight whatever option(s) are applicable

Basic research

Translational research

Clinical research

Patient care

Primary prevention including screening

Rehabilitation

Supportive/palliative care

Other, please explain

2. What is your major source of funding (that constitutes the majority of your budget)? Please highlight whatever option is applicable

Public (money from governmental organisation, insurance)

Private (private investors, NGO's)

Other, please explain

3. Please fill out the table below about the types of performance assessment that your centre participates in.

Please highlight whatever option(s) are applicable for each assessment. Please expand the table if necessary.

| **Name of the organisation doing the performance assessment** | **Is that organisation public(governmental) or private (non governmental)** | **Is the assessment voluntary or mandatory** | **On which level is the performance assessment conducted** | **Which of the following activities are assessed(please chose all applicable)** | **How frequently is this assessment done** | **What is the outcome of this assessment** | **Please provide the standards used for this assessment and/or any other relevant documentation (e.g. reports) that can help us understand this assessment. (use space below to provide a link if applicable)** |
| --- | --- | --- | --- | --- | --- | --- | --- |
| 1 | Public  Private | Voluntary  Mandatory | Regional  National  Other, please explain | Basic research  Translational research  Clinical research  Patient care  Primary prevention including screening  Rehabilitation  Supportive/palliative care  Other, please explain | More than once a year  Once a year  Every two years  Every three years  Every five years  Other, please explain | Directly leads to funding  Directly leads to keeping license on any of the activities  Quality improvement  Other, please explain |  |
| 2 | Public  Private | Voluntary  Mandatory | Regional  National  Other, please explain | Basic research  Translational research  Clinical research  Patient care  Primary prevention including screening  Rehabilitation  Supportive/palliative care  Other, please explain | More than once a year  Once a year  Every two years  Every three years  Every five years  Other, please explain | Directly leads to funding  Directly leads to keeping license on any of the activities  Quality improvement  Other, please explain |  |
| 3 | Public  Private | Voluntary  Mandatory | Regional  National  Other, please explain | Basic research  Translational research  Clinical research  Patient care  Primary prevention including screening  Rehabilitation  Supportive/palliative care  Other, please explain | More than once a year  Once a year  Every two years  Every three years  Every five years  Other, please explain | Directly leads to funding  Directly leads to keeping license on any of the activities  Quality improvement  Other, please explain |  |
| 4 | Public  Private | Voluntary  Mandatory | Regional  National  Other, please explain | Basic research  Translational research  Clinical research  Patient care  Primary prevention including screening  Rehabilitation  Supportive/palliative care  Other, please explain | More than once a year  Once a year  Every two years  Every three years  Every five years  Other, please explain | Directly leads to funding  Directly leads to keeping license on any of the activities  Quality improvement  Other, please explain |  |
| 5 | Public  Private | Voluntary  Mandatory | Regional  National  Other, please explain | Basic research  Translational research  Clinical research  Patient care  Primary prevention including screening  Rehabilitation  Supportive/palliative care  Other, please explain | More than once a year  Once a year  Every two years  Every three years  Every five years  Other, please explain | Directly leads to funding  Directly leads to keeping license on any of the activities  Quality improvement  Other, please explain |  |
| 6 | Public  Private | Voluntary  Mandatory | Regional  National  Other, please explain | Basic research  Translational research  Clinical research  Patient care  Primary prevention including screening  Rehabilitation  Supportive/palliative care  Other, please explain | More than once a year  Once a year  Every two years  Every three years  Every five years  Other, please explain | Directly leads to funding  Directly leads to keeping license on any of the activities  Quality improvement  Other, please explain |  |
| 7 | Public  Private | Voluntary  Mandatory | Regional  National  Other, please explain | Basic research  Translational research  Clinical research  Patient care  Primary prevention including screening  Rehabilitation  Supportive/palliative care  Other, please explain | More than once a year  Once a year  Every two years  Every three years  Every five years  Other, please explain | Directly leads to funding  Directly leads to keeping license on any of the activities  Quality improvement  Other, please explain |  |
| 8 | Public  Private | Voluntary  Mandatory | Regional  National  Other, please explain | Basic research  Translational research  Clinical research  Patient care  Primary prevention including screening  Rehabilitation  Supportive/palliative care  Other, please explain | More than once a year  Once a year  Every two years  Every three years  Every five years  Other, please explain | Directly leads to funding  Directly leads to keeping license on any of the activities  Quality improvement  Other, please explain |  |
| 9 | Public  Private | Voluntary  Mandatory | Regional  National  Other, please explain | Basic research  Translational research  Clinical research  Patient care  Primary prevention including screening  Rehabilitation  Supportive/palliative care  Other, please explain | More than once a year  Once a year  Every two years  Every three years  Every five years  Other, please explain | Directly leads to funding  Directly leads to keeping license on any of the activities  Quality improvement  Other, please explain |  |
| 10 | Public  Private | Voluntary  Mandatory | Regional  National  Other, please explain | Basic research  Translational research  Clinical research  Patient care  Primary prevention including screening  Rehabilitation  Supportive/palliative care  Other, please explain | More than once a year  Once a year  Every two years  Every three years  Every five years  Other, please explain | Directly leads to funding  Directly leads to keeping license on any of the activities  Quality improvement  Other, please explain |  |
