# Supplementary material for: Quality assessments for cancer centers in the European Union
Source: BMC Health Serv Res. 2016 Sep 7;16(1):474. doi: 10.1186/s12913-016-1738-2 (PMC5013566; doi:10.1186/s12913-016-1738-2)
Supplement: Additional file 2: Table 2. — Scope and nature of assessments. This table contains information regarding the Scope (patient, research, combination) and Nature (mandatory or voluntary) of the assessments per country. (DOC 49 kb) [file 12913_2016_1738_MOESM2_ESM.doc]

Additional file 2 Scope and nature of assessments

| **EU member state** | Pure research assessments | | |  | Patient care assessments | | | | Research + Patient care assessments | | | |  |  |
| --- | --- | --- | --- | --- | --- | --- | --- | --- | --- | --- | --- | --- | --- | --- |
|  | **Mandatory** | **Average frequency** | **Voluntary** | **Average frequency** | **Mandatory** | **Average frequency** | **Voluntary** | **Average frequency** | **Mandatory** | **Average frequency** | **Voluntary** | **Average**  **frequency** | **Programs operational** |  |
| Austria | 1 | 2 years | 0 | - | 0 | - | 4 | 2,7 years | 0 | - | 1 | 2 years | 6 |  |
| Croatia* | 0 | - | 0 | - | 1 | 1 year | 0 | - | 0 | - | 0 | - | 1 |  |
| Czech Republic | 1 | - | 1 | Irregular | 0 | - | 1 | 3 years | 2 | 1,5 years | 3 | 3 years | 8 |  |
| Denmark | 0 | - | 1 | 1 year | 0 | - | 0 | - | 0 | - | 0 | - | 1 |  |
| Estonia | 2 | 2 years | 1 | 1 year | 3 | 2,7 year | 1 | 3 year | 0 | - | 0 | - | 7 |  |
| Finland | 0 | - | 5 | 4,3 years | 0 | - | 1 | 2 years | 1 | NC¹ | 2 | 4 years | 9 |  |
| France | 1 | 2 years | 0 | - | 1 | 4 years | 0 | - | 0 | - | 1 | 4 years | 3 |  |
| Germany | 1 | 5 years | 0 | - | 1 | NC¹ | 0 | - | 1 | 3 years | 0 | - | 3 |  |
| Hungary | 0 | - | 0 | - | 1 | OTE³ | 0 |  | 1 | 3 years | 1 | 4 years | 3 |  |
| Ireland* | 0 | - | 0 | - | 0 | - | 1 | 3 years | 0 | - | 0 | - | 1 |  |
| Italy | 0 | - | 1 | 1 year | 1 | 0,3 year | 1 | 3 years | 0 | - | 4 | 2,5 years | 7 |  |
| Lithuania | 0 | - | 0 | - | 5 | 3 years | 0 | - | 1 | NC¹ | 1 | 4 years | 7 |  |
| Netherlands | 0 | - | 0 | - | 2 | 1 year | 1 | 3 years | 0 | - | 2 | 3 years | 5 |  |
| Poland | 0 | - | 0 | - | 2 | MTOY² | 0 |  | 3 | 1,3 years | 1 | 1 year | 6 |  |
| Portugal | 0 | - | 0 | - | 1 | MTOY² | 0 | - | 3 | 3 years | 1 | 3 years | 5 |  |
| Slovenia | 3 | 1 year | 2 | 1,1 years | 5 | 2,6 years | 6 | 1,5 years | 2 | 1 year | 2 | 3 years | 20 |  |
| Spain | 1 | MTOY² | 1 | 1 year | 3 | 1,5 years | 1 | MTOY² | 0 | - | 1 | 4 years | 7 |  |
| United Kingdom | 5 | 4,25 years | 0 | - | 1 | 2 years | 1 | 2 years | 1 | 1 year | 2 | 2,5 years | 10 |  |
| SUBTOTAL | 15 |  | 12 |  | 27 |  | 18 |  | 15 |  | 22 |  |  | |
| TOTAL | 27 | | |  | 45 | | |  | 37 | | |  | 109 | |

¹ NC: Not Clear
² MTOY: More than Once a Year
³ OTE: One Time Event
