# Supplementary material for: Quality assessments for cancer centers in the European Union
Source: BMC Health Serv Res. 2016 Sep 7;16(1):474. doi: 10.1186/s12913-016-1738-2 (PMC5013566; doi:10.1186/s12913-016-1738-2)
Supplement: Additional file 3: Table 3. — Overview of assessments including details per country. This table contains all assessments included in this study, divided per country and category (patient, research, combination). (DOC 124 kb) [file 12913_2016_1738_MOESM3_ESM.doc]

**Additional file 3 Assessments listed per country**

| **Country** | **Care** (year in which assessment was first performed and frequency) | Assessment details | **Research** (year in which assessment was first performed and frequency) | Assessment details | **Mixed**  (year in which assessment was first performed and frequency) | Assessment details | |
| --- | --- | --- | --- | --- | --- | --- | --- |
| Lithuania | National Health Insurance Fund (2003) Every three years <http://www.vaspvt.gov.lt/en/node/540> | Mandatory License National Public |  |  | National Audit Office of Lithuania (1990) If necessary. According to this institution annual plan <http://www.vkontrole.lt/default_en.aspx> | Mandatory QI National Public | |
|  | National Health Insurance Fund (2012) If necessary. According to this institution annual plan <http://www.vlk.lt/sites/en> | Mandatory QI* National Public |  |  | OECI (Organization of European Cancer Institutes) (2013) Every four years | Voluntary QI International Private | |
|  | Ministry of Health of the Republic of Lithuania (1990) If necessary. According to this institution annual plan <http://www.sam.lt/go.php/lit/English> | Mandatory QI National Public |  |  |  |  | |
|  | State Social Insurance Fund Board  (1990) If necessary. According to this institution annual plan <http://www.sodra.lt/en> | Mandatory Funding+QI National Public |  |  |  |  | |
|  | Radiation Protection Centre  (1997) If necessary. According to this institution annual plan <http://www.rsc.lt/index.php/pageid/445> | Mandatory QI National Public |  |  |  |  | |
| Estonia | Estonian Ministry of Social Affairs (2001) Every five years | Mandatory QI National Public | Estonian Science Agency (2001) Once a year | Mandatory Funding National Public |  |  | |
|  | Estonian Sick Foundation (2001) Once a year | Mandatory Funding National Public | LabQuality (2001) Once a year | Voluntary QI Regional Private |  |  | |
|  | Estonian Drug Agency (2006) Every two years | Mandatory License+QI National Public | Estonian Center of Accreditation (2010) Every three years | Mandatory License+QI National Public |  |  | |
|  | IAEA (2009/2012) Every three years | Voluntary QI Regional |  |  |  |  | |
| France | National Health Authority (HAS)  Every four years | Mandatory License+QI National Public | Evaluation agency for research and higher education (AERES)  Every two years <http://www.aeres-evaluation.com/Evaluation/Evaluation-of-research-units/Evaluation-principles> | Mandatory Funding National Public | OECI (Organization of European Cancer Institutes) Every four years | Voluntary QI International Private | |
| Italy | Institutional Accreditation (2003/2009) More than once a year (four mounth) | Mandatory License+QI Regional/ National Public | European federation for Immunogenetics (2008) Once a year | Voluntary QI International Private | ISO 9001:2008 (2002) Once a year | Voluntary Funding+QI National/ International Private | |
|  | ENETS (European Neuroendocrine Tumor Society) (2010) Every three years | Voluntary QI International Private |  |  | JACIE (2010/2013) Every two years <http://www.jacie.org/standards/interim-standards> | Voluntary License+QI National/ International Private | |
|  |  |  |  |  | Accreditation Canada International (2010) Every three years | Voluntary License+QI International Private | |
|  |  |  |  |  | OECI (2014) Every four years | Voluntary QI International Private | |
| Germany | Clinical process certification by Deutsche Krebsgesellschaft (DKG)  http://www.krebsgesellschaft.de/wub_zertifizierte_zentren_english,237958.html | National Public | Helmholtz Gemeinschaft (2007) Every five years | Mandatory Funding National Public | Deutsche Krebshilfe (DKH)  5^th^ Call Interdisciplinary Oncology Centers of Excellence (2009) Every three years http://www.krebshilfe.de/wir-foerdern/ausschreibungen.html | Mandatory Funding National Public | |
| Portugal | ACSS (Administração Central de Sistemas de Saúde) (2012) More than once a year | Mandatory Funding+ License+QI National Public |  |  | OECI  (2009) Every four years | Voluntary QI International Private | |
|  |  |  |  |  | CHKS (Caspe Healthcare Knowledge System) (2004) Every two years | Voluntary QI International Private | |
|  |  |  |  |  | FCT (Fundação para a Ciência e Tecnologia) (2007) Every three years | Mandatory Funding + License+QI  National Public | |
|  |  |  |  |  | APCER (Associação Portuguesa de Certificação) (2007) Every three years | Voluntary QI National Private | |
| Hungary | International Atomic Energy Agency (2006) One time event | Voluntary  QI International Public |  |  | Dekra Certification Kft.  (based on ISO) (2001) Every three years | Mandatory QI National  Private | |
|  |  |  |  |  | OECI accreditation (2008) Every four years | Voluntary QI International Private | |
| UK | JACIE Every two years <http://www.jacie.org/> | Mandatory License+QI International Private | Research Excellence Framework (REF)  ? | Mandatory Funding National Public | NHS England (National peer review programme) Once a year | Mandatory License+QI National Public | |
|  | Care Quality Commission (CQC) Every two years <https://www.gov.uk/government/uploads/system/uploads/attachment_data/file/227075/IRMER_regulations_2000.pdf> | Voluntary QI National Public | Scientific Reviews of Individual Institutes and Individual Assessments Every five years | Mandatory Funding National Public | British Standards Institute (ISO 9001) Once a year <http://www.bsigroup.co.uk/en-GB/iso-9001-quality-management/> | Voluntary QI National Public/Private | |
|  |  |  | Cancer Research UK Centre Review  and Experimental Cancer Medicine Centre Review Every five years | Mandatory Funding National | Organisation of European Cancer Institutes (OECI) Every four years | Voluntary QI International  Private | |
|  |  |  | Biomedical Research Council Every five years | Mandatory Funding National Public |  |  | |
|  |  |  | Human Tissue Authority (HTA) Every two years <http://www.hta.gov.uk/_db/_documents/Q&S_Human_Application_Regs_2007.pdf> | Mandatory License National Public |  |  | |
| Spain | SEAP (Spanish Society of Pathology) (2012) More than once a year | Voluntary QI National Private | ANEP (National Agency for Projects Evaluation) (2002) More than once a year | Mandatory Funding National Public | OECI (2010) Every four years | Voluntary QI International Private | |
|  | Plan of Inspection Valencia Health Agency (1998) By express request of the competent authority | Mandatory Funding+ License+QI  Regional/ National Public | EMQN  (The European Molecular Genetics  Quality Network) (2009) Once a year | Voluntary QI International Private |  |  | |
|  | Certification for Nuclear Medicine (2002) Once a year | Mandatory QI National/ International Public |  |  |  |  | |
|  | Department of Health. Inspection for Blood Transfusion Department (1997) Every two years | Mandatory License Regional Public |  |  |  |  | |
| Poland | NFZ  National Health Service/Insurance Found More than once a year www.nfz.gov.pl | Mandatory Funding+ License National Public |  |  | National Atomic Energy Agency  Once a year www.paa.org | Voluntary QI National Private | |
|  | SANEPID More than once a year | Mandatory License+QI Regional Public |  |  | ISO Certification and/or validation Every two years | Mandatory Funding+ License+QI Regional Public | |
|  |  |  |  |  | Local Government More than once a year | Mandatory Funding+ License National Public | |
|  |  |  |  |  | Ministry of Health Once a year | Mandatory QI National Public | |
| Slovenia | European External Quality Assessment (EQA) program for testing biomarker mutations in colorectal cancer (2011) Once a year | Voluntary QI International | The European Molecular Genetics Quality Network (EMQN) (2009) Once a year | Voluntary QI International | Ministry of health (2007)  Every five years | Voluntary Funding National Public | |
|  | RCPAQAP- The Royal College of Pathologists of Australasia Quality Assurance Programs (2011) Once a year <http://www.rcpaqap.com.au/> | Voluntary QI International Private | Ministry of agriculture and environment (1970) According to their risk assessment – usually once per year | Mandatory License National Public | National health insurance company (ZZZS) (2004) Every year | Voluntary License National Public | |
|  | Ministry of Health (2010) Every five years | Mandatory License National Public | NEQAS (Great Britain)  (2012) More than once a year | Voluntary QI International Private | QUATRO  audit: in 2011 (2011) Only once | Mandatory License National Private | |
|  | ESMO Designated Centers of Integrated Oncology & Palliative Care  (2010) Every three years <http://www.esmo.org/Patients/Designated-Centres-of-Integrated-Oncology-and-Palliative-Care> | Voluntary QI International Private | NordiQC  (2012) More than once a year www.nordiqc.org | Voluntary Funding+ License+QI Public | External  Audit by ZVD  (Institute of occupational safety)  Once a year <http://www.uvps.gov.si/en/> | Mandatory License+QI International Private | |
|  | THE BOWEL  CANCER SCREENING PROGRAMME  EQA SCHEME (2012) Once a year www.virtualpathology.leeds.ac.uk/eqa | Mandatory QI International Public | UK NEQUAS (2014) More than once a year (every month) www.uknequas.org | Voluntary QI International Public |  |  | |
|  | National board for quality assurance in cervical screening (2008) Once a year | Mandatory QI National Public |  |  |  |  | |
|  | UK Nequas (2001) More than once a year | Voluntary QI International Public |  |  |  |  | |
|  | INSTAND e.V.  Dusseldorf, Germany  (2013) Once a year | Mandatory QI International |  |  |  |  | |
|  | ESPEN  (2009) Once a year [www.nutritionday.org](http://www.nutritionday.org/) | Voluntary QI National Public |  |  |  |  | |
|  | Boehringer Ingelhaim  (2010)  Once | Voluntary License International Private |  |  |  |  | |
|  | Republic of Slovenia  Ministry of Health  (2010) every five years  http://www.mz.gov.si/si/delovna_podrocja/zdravstveno_varstvo/koordinacija_sistema_zdravstvenega_varstva/laboratoriji_za_izvajanje_preiskav_na_podrocju_laboratorijske_medicine/ | Mandatory QI National Public |  |  |  |  | |
| Croatia | Croatian National Institute of  Public Health (2006) | Mandatory QI National Public |  |  |  |  | |
| The Netherlands | IGZ  Once a year <http://www.igz.nl/onderwerpen/curatieve-gezondheidszorg/ziekenhuizen/index.aspx> | Mandatory QI National Public |  |  | OECI (2011) Every four years [http://oeci.selfassessment.nu/userfiles/file/empty%20qualitativ%20questionnaire.pdf](http://oeci.selfassessment.nu/userfiles/file/empty qualitativ questionnaire.pdf) | Voluntary QI International  Private | |
|  | DNV  (2013) Once a year | Mandatory QI National Private |  |  | JACIE Every two years | Voluntary License+QI International Private | |
|  | ENETS (European Neuroendocrine Tumor Society) Every three year | Voluntary QI International Private |  |  |  |  | |
| Ireland | IMPACT Every three years [www.ces-vol.org.uk](http://www.ces-vol.org.uk/) | Voluntary QI National Private |  |  |  |  | |
| Czech Republic | Joint Accreditation Commission  (Czech Republic)  (Spojená akrediatační komise, o.p.s.)  Every three years <http://www.sakcr.cz/cz-top/o-nas/> | Voluntary Funding+ License+QI  National Private | State Institute for Drug Control (Czech: Státní ústav pro kontrolu léčiv, SÚKL) [www.sukl.cz](http://www.sukl.cz/) | Mandatory QI National Public | Joint Commission International (USA) Every three years  [http://www.jointcommissioninternational.org](http://www.jointcommissioninternational.org/) | Voluntary Funding+ License+QI  International Private | |
|  |  |  | State Office for Nuclear Safety (Czech: Státní ústav pro jadernou bezpečnost) (2010) [www.sujb.cz](http://www.sujb.cz/) | Voluntary License National Public | International Atomic Energy Agency (IAEA)  <http://www-pub.iaea.org/MTCD/publications/PDF/Pub1297_web.pdf> | Voluntary QI International Public | |
|  |  |  |  |  | Czech Accreditation Institute  (Czech name: Český institut pro akreditaci, o.p.s.)  Once a year [www.cia.cz](http://www.cia.cz/) | Mandatory Funding+ License+QI  National Private | |
|  |  |  |  |  | National authorization center for clinical laboratories  (Czech name: Národní autorizační středisko pro klinické Reportlaboratoře, NASKL)  Every two years <http://www.naskl.cz/> | Mandatory  Funding+ License+QI National Public | |
|  |  |  |  |  | State Office for Nuclear Safety (Czech: Státní ústav pro jadernou bezpečnost)  Once a year | Mandatory ??? National Public | |
| Denmark |  |  | International Scientific Advisory Board (2014) Once a year | Voluntary QI International Private |  |  | |
| Finland | JACIE (Joint Accreditation Committee – ISCT)  (2006) Every two years <http://www.jacie.org/document-centre> | Voluntary QI International Public | University of Helsinki (2007) Every seven years <http://www.helsinki.fi/arviointi2010-2012/eng/> | Voluntary QI Regional Public | Scientific Advisory Board  (of the HUCH CCC)  (2015) | Mandatory License+QI International Private | |
|  |  |  | Academy of Finland (1995) Every two years <http://www.aka.fi/Tiedostot/Tiedostot/Huiput2009/CoE_procedure_eng.pdf> | Voluntary Funding National Public | EUHANET  (European Haemophilia Network) (2014) <http://www.euhanet.org/MappedCentres.aspx> | Voluntary QI International Public | |
|  |  |  | Academy of Finland and  Swedish Research Council (2008) <https://publikationer.vr.se/en/product/clinical-research-in-finland-and-sweden/> | Voluntary QI National Public | OECI (2014) Every four years <http://oeci.selfassessment.nu/cms/node/53> | Voluntary QI International Public | |
|  |  |  | Academy of Finland (1997) Every three years [http://www.aka.fi/en-GB/A/Decisions-and-impacts/The-state-of-scientific-research-in-Finland/The-state-of-scientific-research-in-Finland-20121/Laake--ja-terveystieteet/#el%C3%A4in](http://www.aka.fi/en-GB/A/Decisions-and-impacts/The-state-of-scientific-research-in-Finland/The-state-of-scientific-research-in-Finland-20121/Laake--ja-terveystieteet/#el‚in) | Voluntary QI National Public |  |  | |
|  |  |  | Biomedicum Helsinki (2004)  Every five years | Voluntary Funding Regional Public |  |  | |
| Austria | Doc-Cert Every three years  <http://www.doc-cert.com/64/brustgesundheitszentren/ablauf-der-zertifizierung> | Voluntary License+QI National/ International Private | BASG /AGES Every two years  https://www.ris.bka.gv.at/GeltendeFassung.wxe?Abfrage=Bundesnormen&Gesetzesnummer=20005698&ShowPrintPreview=True  http://www.basg.gv.at/bundesamt-fuer-sicherheit-im-gesundheitswesen-basg/ | Mandatory QI National/ International Public | EFQM Quality Austria Every two years  http://www.qualityaustria.com/  http://www.qualityaustria.com/index.php?id=2741&L=1 | Voluntary QI National/ International Private |  |
| ISO 9001:2008 Quality Austria Every year  http://www.qualityaustria.com/index.php?id=2238 | Voluntary QI National/ International Private |  |  |  |  |  |
| Pain-cert certkom  <http://www.paincert.org/index.php?id=2>  http://www.certkom.com/ | Voluntary QI National/ International Private |  |  |  |  |  |
| ONR 49000ff and ISO 31000 MC&T Every four years  http://www.mc-t.at/risikomanagement.php | Voluntary QI National/ International Private |  |  |  |  |  |
